# Identifying microRNAs and Their Editing Sites in *Macaca mulatta*

**DOI:** 10.3390/cells8070682

**Published:** 2019-07-05

**Authors:** Qingyi Wang, Zhigang Zhao, Xiaotuo Zhang, Chenyu Lu, Shuchao Ren, Shipeng Li, Junqiang Guo, Peiran Liao, Bingbing Jiang, Yun Zheng

**Affiliations:** 1 Faculty of Information Engineering and Automation, Kunming University of Science and Technology, Kunming 650500, China; 2 Yunnan Key Lab of Primate Biomedicine Research, Institute of Primate Translational Medicine, Kunming University of Science and Technology, Kunming 650500, China; 3 State Key Laboratory of Genetic Engineering, Collaborative Innovation Center for Genetics and Development, Institute of Plant Biology, School of Life Sciences, Fudan University, Shanghai 200438, China

**Keywords:** miRNA, microRNA editing, *Macaca mulatta*, small RNA-seq, bioinformatics

## Abstract

MicroRNAs (miRNAs) are small non-coding RNAs that are critical in post-transcriptional regulation. *Macaca mulatta* is an important nonhuman primate that is often used in basic and translational researches. However, the annotation of miRNAs in *Macaca mulatta* is far from complete, and there are no reports of miRNA editing events in *Macaca mulatta*, although editing may affect the biogenesis or functions of the miRNAs. To improve miRNA annotation and to reveal editing events of miRNAs in *Macaca mulatta*, we generated 12 small RNA profiles from eight tissues and performed comprehensive analysis of these profiles. We identified 479 conserved pre-miRNAs that have not been reported in *Macaca mulatta* and 17 species specific miRNAs. Furthermore, we identified 3386 editing sites with significant editing levels from 471 pre-miRNAs after analyzing the 12 self-generated and 58 additional published sRNA-seq profiles from 17 different types of organs or tissues. In addition to 16 conserved A-to-I editing sites, we identified five conserved C-to-U editing sites in miRNAs of *Macaca mulatta* and *Homo sapiens*. We also identified 11 SNPs in the miRNAs of *Macaca mulatta*. The analysis of the potential targets of 69 miRNAs with editing or mutation events in their seed regions suggest that these editing or mutation events severely changed their targets and their potential functions. These results significantly increase our understanding of miRNAs and their mutation/editing events in *Macaca mulatta*.

## 1. Introduction

MicroRNAs are small non-coding RNAs with about 22 nucleotides that could repress their target mRNAs [1]. The miRNA system is conserved from worms to mammals [2,3], which indicates its important functions. MiRNAs are involved in many biological processes including cell cycle, differentiation, development, metabolism, and so on [4,5,6,7,8,9]. Recent studies have emphasized the essential roles of miRNAs in diverse diseases [10,11,12,13].

Some miRNAs may be edited in multiple ways during their biogenesis processes [14,15,16,17,18,19,20,21,22,23,24,25,26]. These editing events could either change the nucleotide of mature miRNAs, such as Adenosine-to-Inosine (A-to-I) editing [27], or change the secondary structures of pre-miRNAs and processing of mature miRNAs [26,28,29,30]. Adenosine-to-Inosine (A-to-I) editing is performed by ADAR (adenosine deaminase) on the double-stranded RNAs [14,25] to convert an adenosine residue into an inosine residue [18,24,25]. Inosine residue converted from adenosine in RNA is read as guanosine during reverse transcription for RNA-seq [17,18,24,25,31].

C-to-U editing is performed by apolipoprotein B mRNA editing catalytic polypeptide-like (APOBEC) enzymes [32,33,34]. C-to-U editing is widely existing in plant mitochondria and chloroplasts [33]. Recently, it was reported that some animal miRNAs have C-to-U editing sites as well [32,35,36].

Many miRNAs were modified at their 3’ end to add non-templated nucleotides, especially U and A [17,20,26,37]. Generally, 3’-U and 3’-A induces and prevents the degradation of miRNAs, respectively [38]. 3’-A may also affect the loading of mature miRNAs to RNA-induced silencing complex (RISC) [20]. Since Single Nucleotide Polymorphisms (SNPs) also change the nucleotide sequences of miRNAs, the transcription and processing of pri-miRNAs and pre-miRNAs, maturation of mature miRNAs, and/or complementarity to mRNAs could be affected by SNPs [39,40]. The SNPs and abnormal editing events in miRNAs have been linked to severe diseases [41,42].

*Macaca mulatta* is an important model primate animal and widely used in basic and translational research as a nonhuman primate [43]. However, the annotation of miRNAs in *Macaca mulatta* is far from complete and accurate. There are only 626 annotated pre-miRNAs of *Macaca mulatta* in the latest version of miRBase (v22) [44], which is much less than the 1917 in *Homo sapiens*, although both two species are primates. The Ensembl database (version Mmul_8.0.1) contains 1951 annotated miRNAs, of which 1331 ones did not include the annotation of mature miRNAs. The editing events in miRNAs of *Macaca mulatta* are still largely unexplored.

With the development of high throughput sequencing technologies recently, the small RNAs, including miRNAs, edited miRNAs, miRNAs originated from mutated DNAs, and fragments of mRNA degradation products, in the samples could be obtained with high throughput sequencing profiles of small RNAs. The small RNA sequencing (sRNA-seq) profiles thus could be used to identify miRNA genes in genome and their editing sites.

Therefore, to improve the annotation of miRNAs in *Macaca mulatta* and to comprehensively identify Mutation/Editing (M/E) sites in miRNAs of *Macaca mulatta*, we sequenced small RNAs for 12 samples from eight different tissues of *Macaca mulatta* and performed comprehensive identification of both conserved and non-conserved miRNAs. Furthermore, to identifying editing sites in miRNAs of *Macaca mulatta* in different organs or tissues, we then collected 58 published sRNA-seq profiles, and systematically analyzed 70 sRNA-seq profiles (including the 12 self-generated and 58 published ones) from 17 different types of organs or tissues. To examine conservation of editing sites in miRNAs of *Macaca mulatta*, the identified M/E sites were compared to the miRNA editing sites in *Homo sapiens*. Since the M/E sites in seed regions may change the potential targets of miRNAs, we examined the targets of miRNAs with M/E sites in their seed regions, and the potential functions of the original and edited/mutated miRNAs by analyzing the enriched Gene Ontology (GO) terms and KEGG pathways of their targets.

## 2. Results and Discussion

### 2.1. Collection of Tissue Samples and sRNA-Sequencing

We collected twelve samples from the eight different tissues of two wild type *Macaca mulatta* animals, namely muscle (2), liver (1), kidney (2), small intestine (2), large intestine (2), lung (1), spinal cord (1), and occipital lobe (1) without treatments. The small RNAs of obtained samples were sequenced with Illumina HiSeq 2000 sequencer, and generated at least 20 million small RNA reads for each of these 12 samples (Table S1). We aligned these 12 sRNA-seq profiles to different categories of molecules and found that most of the obtained sRNA sequencing reads could be aligned to the genome (see Table S2 and Figure S1b), suggesting the qualities of the obtained profiles are good. These 12 sRNA-seq profiles were available at the NCBI GEO database under the series accession number GSE124417.

### 2.2. Identifying Conserved miRNAs of Macaca mulatta

**Figure 1:**
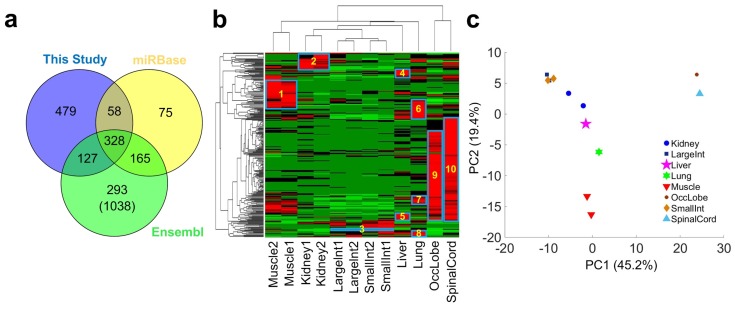
A summary of the identified conserved miRNAs and analysis of their expression patterns in different tissues. (**a**) The number of conserved miRNAs identified in this study and those reported in the miRBase (v22) and the Ensembl database (version Mmul_8.0.1). The 1038 miRNAs in parenthesis annotated in the Ensembl database lack clear annotations of mature miRNAs. (**b**) The bi-directional clustering analysis using the normal expression levels of selected conserved miRNAs in the 12 self-generated sRNA-seq profiles. The miRNAs in the Rectangles 1–10 are regarded as highly expressed miRNAs in specific tissues or organs. The detailed list of miRNAs in Rectangles 1–10 are given in Sets 1–10 in Table S5, respectively. (**c**) The Principle Component Analysis using the normal expression levels of selected conserved miRNAs in the 12 self-generated sRNA-seq profiles.

To obtain a better annotation of miRNAs in *Macaca mulatta*, we first downloaded the 626 miRNAs of *Macaca mulatta* in the miRBase (v22) [45] and 1951 transcripts annotated as miRNAs in the Ensembl database (version Mmul_8.0.1). Then, by using a computational pipeline proposed previously [46] (see Figure S1a, and details in Materials and Methods), we totally found 992 conserved pre-miRNAs (Figure 1a). These three sets were compared based on their genomic loci (as shown in Figure 1a) and merged to obtain a list of 1525 pre-miRNAs (Table S3). There are 1331 pre-miRNAs that are only reported in Ensembl database and do not have mature miRNA annotation. Therefore, we aligned the mature miRNAs in the miRBase (v22) to these 1331 pre-miRNAs and found mature miRNAs for 293 of these 1331 pre-miRNAs. It still needs more evidence to verify whether the 1038 remaining pre-miRNAs in the Ensembl database are real miRNAs.

### 2.3. Expression Patterns of Conserved miRNAs in Different Tissues/Organs

The RPTM values of 2756 mature miRNAs in the 1525 pre-miRNAs were calculated using a computational pipeline introduced previously [46]. After removing miRNAs with very low abundances, 320 miRNAs were selected for further analysis (as listed in Table S4). Next, we performed hierarchical clustering and Principle Component Analysis using the RPTM values of selected miRNAs (see Materials and Methods). As shown in Figure 1b, some miRNAs were highly expressed in specific organs or tissues. For example, it had been reported that miR-1 and miR-133 form a highly conserved miRNA cluster in the genome and are critical regulators of skeletal muscle proliferation and differentiation [47]. Consistent with this work, our results show that mmu-miR-1a/b-3p and mml-miR-133a/b/c-3p are highly expressed in the two muscle samples (in Rectangle 1 of Figure 1b and Set 1 of Table S5). mmu-miR-10a-5p and mml-miR-10b-5p are highly expressed in the two kidney samples, which is consistent with previous results of highly expressed miR-10a in mouse kidney [48]. In addition, as shown in Figure 1b, there are much more highly expressed miRNAs in the two neural tissue samples, i.e., occipital lobe and spinal cord.

As shown in Figure 1b,c, samples of the same or similar types of organs/tissues were correctly grouped together, suggesting that these conserved miRNAs carry important molecular information that are specific to different types of organs and/or tissues.

### 2.4. Identifying Species Specific miRNAs of Macaca mulatta

**Figure 2:**
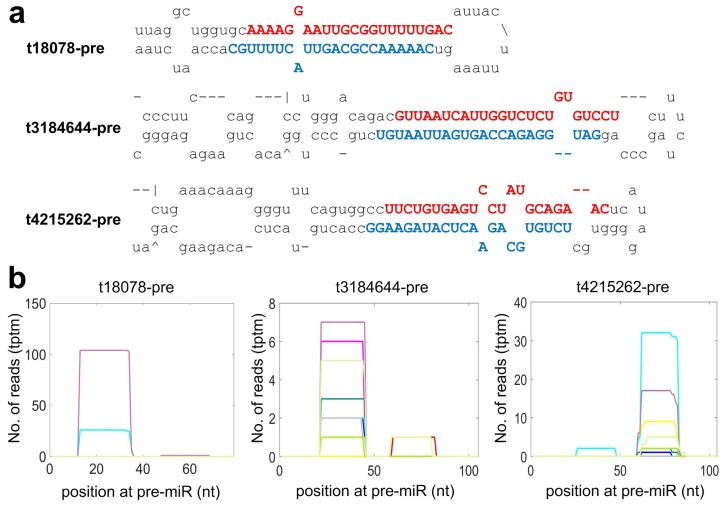
Three of the species specific miRNAs of *Macaca mulatta* identified in this study. (**a**) The secondary structure of precursors of identified miRNAs. The blue and red regions in the precursors represent the mature miRNAs that were sequenced in the 12 self-generated sRNA-seq profiles. (**b**) The distributions of sequencing reads on the pre-miRNAs in (**a**).

As shown in Figure S1c, in addition to the conserved miRNAs, we also identified 17 species specific miRNAs (as listed in Table S6) by using a set of stringent criteria for annotating miRNAs [46,49]. For examples, three of the 17 species specific miRNAs are shown in Figure 2. As shown in Figure 2a, the mature miRNAs of both strands of the pre-miRNAs were detected in the 12 self-generated sRNA-seq profiles with a 2 nt overhang at the 3’ end, which is one of typical characteristics of miRNAs cleaved by Dicer. The distributions of sequencing reads on these three miRNAs also demonstrated clear accumulations in the regions of real mature miRNAs (see Figure 2b). Other species specific miRNAs in Table S6 also had similar distributions of sequencing reads as those in Figure 2b.

### 2.5. Summary of Mutation and/or Editing Sites in miRNAs of Macaca mulatta

To have a comprehensive view of the miRNA editing events of *Macaca mulatta*, we collected 58 public sRNA-seq profiles from different tissues or organs, and analyzed 70 (the 12 self-generated and 58 public) sRNA-seq profiles (as listed in Table S7) with the MiRME pipeline using the default settings [36] (see Figure S1d, and details in Materials and Methods). After combining the results from the 70 sRNA-seq profiles, we totally obtained 58488 M/E sites in the conserved miRNAs of *Macaca mulatta*, of which 3386 sites in 471 pre-miRNAs had significant editing levels in at least one of the 70 samples selected (as listed in Table S8). In addition to conserved miRNAs, we also found 19 M/E sites with significant editing levels, i.e., thirteen 3’-A, four 3’-U and two SNP sites, in species specific miRNAs (as listed in Table S9).

**Figure 3:**
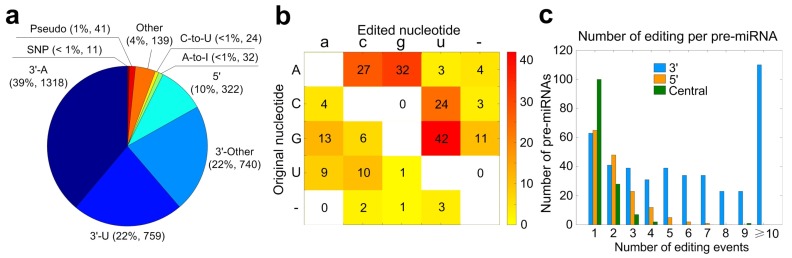
A summary of the identified miRNA mutation and editing sites of *Macaca mulatta*. (**a**) The categories of significant M/E sites in miRNAs. (**b**) The numbers of different types of editing events that do not happen at the 5’ or 3’ end of mature miRNAs. (**c**) The distribution of the numbers of pre-miRNAs with different numbers of 5’- and 3’-editing, and Central editing sites, i.e., editing sites that do not happen at either ends of mature miRNAs.

As shown in Figure 3a, we then classified the 3386 significant M/E sites based on their positions at pre-miRNAs or mature miRNAs into nine different categories, i.e., 3’-A, 3’-U, 3’-Other, 5’ site, A-to-I, C-to-U, Other, SNP and Pseudo site (see [36] for more information of different types of editing sites). After comparing to the results of *Homo sapiens* [36], we found that the percentages of 3’-A and 3’-Other were much larger than those in *Homo sapiens* brain tissues or cell lines. However, the percentages of 3’-U and 5’ sites were intensively decreased.

Next, we examined different types of editing not at the either ends of mature miRNAs. As shown in Figure 3b, there were 32 significant A-to-I and 24 C-to-U editing site, respectively. Meanwhile, our results show that there were many G-to-U and A-to-C sites in miRNAs of *Macaca mulatta* (see Figure 3b), which is very different from those found in *Homo sapiens* brain samples [36].

Some miRNAs may have multiple 3’ editing sites. However, there are only one or two Central editing sites on the same pre-miRNAs (Figure 3c). The number of miRNAs with 5’ editing site in the selected *Macaca mulatta* samples was much smaller than those in *Homo sapiens* brains (Figure 3c).

In summary, our results suggest that the most editing events in miRNAs of *Macaca mulatta* are also happening at the 3’ end of mature miRNAs. A-to-I and C-to-U editing are two main editing types in the central regions of miRNAs, but there could be other editing types in miRNAs of *Macaca mulatta*.

### 2.6. A-to-I Editing Sites in miRNAs of Macaca mulatta

**Figure 4:**
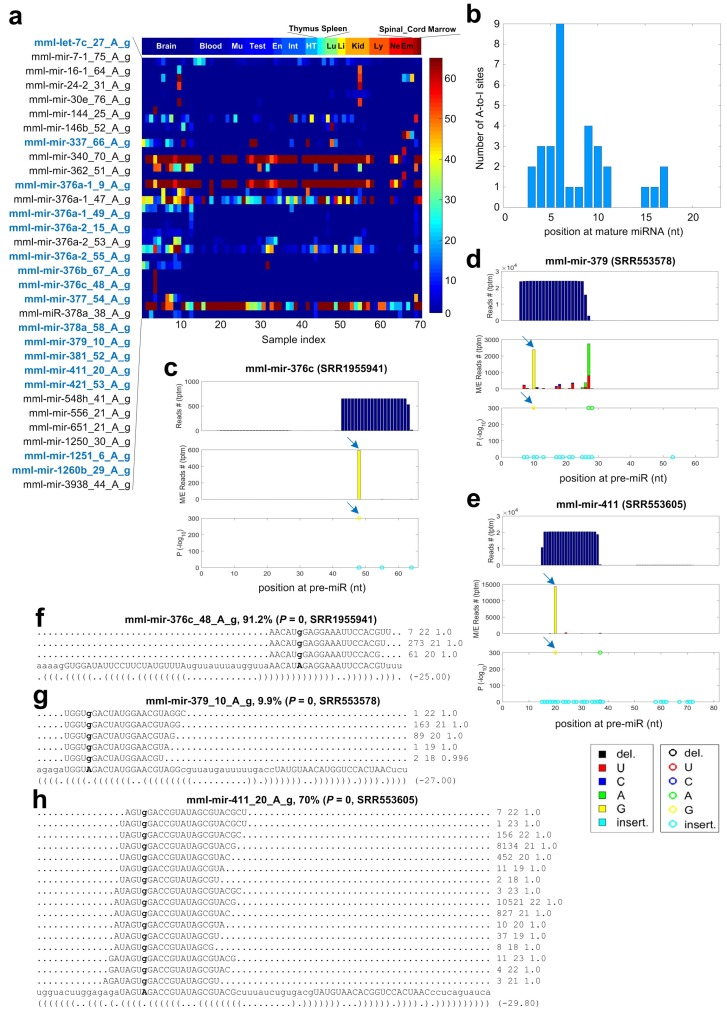
The details of 32 A-to-I editing sites identified in miRNAs of *Macaca mulatta*. (**a**) The editing levels of the 32 A-to-I editing sites in the 70 selected sRNA-seq profiles. The Sample Index of the x-axis is the same as serial in Table S7. The tissues from left to right are Brain, Blood, Muscle (Mu), Testis (Test), Endometrium (En), intestine (Int), Heart (HT), Thymas, Spleen, Lung (Lu), Liver (Li), Kidney (Kid), Lymphoblastoid cell line (Ly), Neural cells (Ne), embryonic stem cells (Em), Spinal cord (Spinal Cord) and bone marrow (Marrow). The sites with blue bold names are conserved in *Homo sapiens*. (**b**) The numbers of A-to-I editing sites at different positions of the mature miRNAs. (**c**) The MiRME map of mml-mir-376c_48_A_g in one of the brain samples selected (SRR1555941). (**d**) The MiRME map of mml-mir-379_10_A_g in one of the brain samples selected (SRR553578). (**e**) The MiRME map of mml-mir-411_20_A_g in one of the brain samples selected (SRR553605). (**f**) The details of mml-mir-376c_48_A_g in SRR1555941. (**g**) The details of mml-mir-144a_32_A_g in SRR1658345. The details of hsa-mir-378d-1_46_A_g in SRR5398637. (**h**) The details of mml-mir-19b-1_64_C_u in SRR1048281. In (**f**–**h**), the edited nucleotides are shown in bold face.

As shown in Figure 4a and Table S9, we carefully examined the 32 A-to-I editing sites with significant editing levels in miRNAs of *Macaca mulatta*. Twenty-seven of these 32 A-to-I editing sites were detected in multiple samples, suggesting that these sites are not random noises. By comparing the positions of editing sites in mature miRNAs to the A-to-I editing sites in miRNAs of *Homo sapiens* [36,50], we found that 16 of the 32 identified A-to-I editing sites are conserved in *Homo sapiens* (see sites with bold blue names in Figure 4a and Table S10). As shown in Figure S2, the same positions of the mature miRNAs have significant A-to-I editing, although the pre-miRNAs in *Macaca mulatta* and *Homo sapiens* may be of different lengths. Four of these 16 conserved A-to-I editing sites, i.e., mml-mir-376a-1_49_A_g, mml-mir-376a-2_55_A_g, mml-mir-376c_48_A_g and mml-mir-1260b_29_A_g, have very high editing levels in many different tissues and samples. Furthermore, six A-to-I editing sites in the miR-376 family are highly conserved in *Homo sapiens*, *Macaca mulatta* and *Mus musculus* [27,36].

As shown in Figure 4b, these A-to-I editing sites prefer to locate in the regions from the fourth to tenth nucleotides of mature miRNAs. Similar to the UAG motif beside A-to-I editing sites in human miRNAs [18,24,36], we found that the 5’ and 3’ nucleotides beside the 32 A-to-I editing sites have a weak preference of U and G, respectively (Figure S3).

As examples, three A-to-I editing sites are shown in Figure 4c–e, respectively, and the reads supporting these sites are shown in Figure 4f–h, respectively. As reported previously [27,36], mml-mir-376c_48_A_g is a conserved editing sites. This site has a very high editing level in a brain pituitary gland sample (SRR1555941), as shown in Figure 4c,g.

Eighteen of these 32 A-to-I editing sites locate in the seed regions of mature miRNAs, which suggests that these A-to-I editing events may lead to modifications of targets of miRNAs. We present the analysis of targets of edited miRNAs in the following.

### 2.7. C-to-U Editing Sites in miRNAs of Macaca mulatta

**Figure 5:**
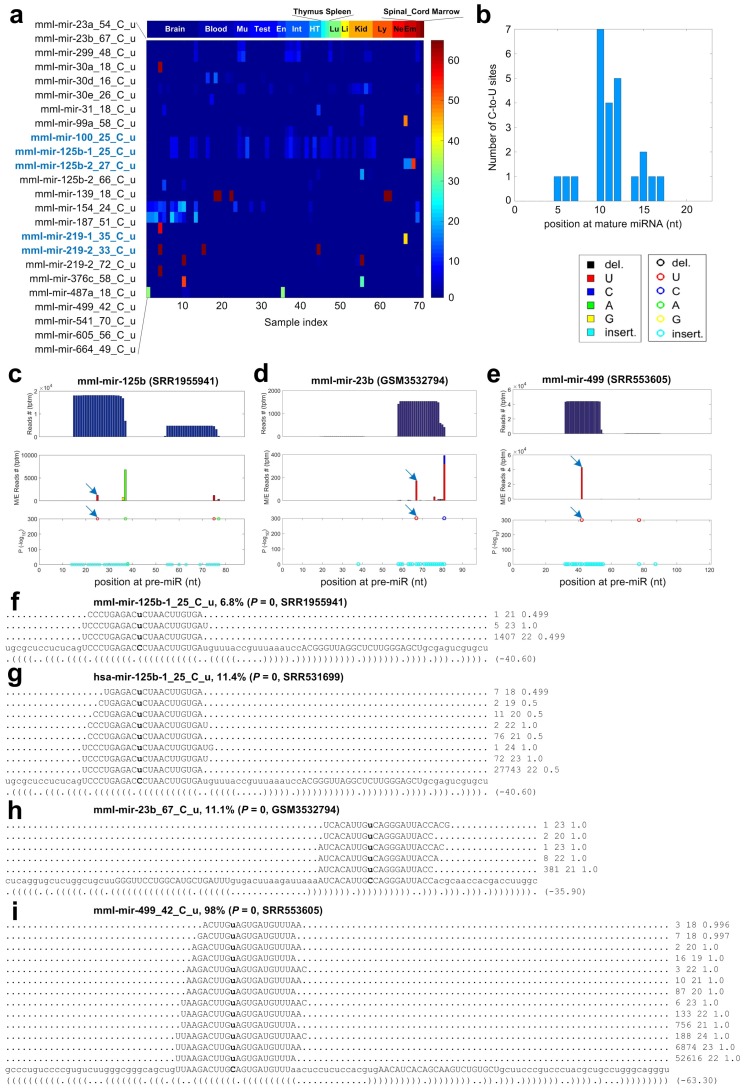
The details of 24 C-to-U editing sites identified in miRNAs of *Macaca mulatta*. Legends are the same as those in Figure 4. (**a**) The editing levels of the 24 C-to-U sites in the 70 selected sRNA-seq profiles. (**b**) The numbers of C-to-U sites at different positions of mature miRNAs. (**c**) The MiRME map of mml-mir-125b-1 in one of the brain samples selected (SRR1955941). (**d**) The MiRME map of mml-mir-23b in one of the muscle samples selected (GSM3532794). (**e**) The MiRME map of mml-mir-499 in one of the brain samples selected (SRR553605). (**f**) The details of mml-mir-23b_67_C_u in GSM3532794. (**g**) The details of hsa-mir-125b-1_25_C_u in a human glioma sample (SRR5874470). (**h**) The details of mml-mir-187_51_C_u in SRR5874470. (**i**) The details of mml-mir-499_42_C_u in SRR553605.

As shown in Figure 3b and Table S11, we identified 24 C-to-U editing sites in miRNAs of *Macaca mulatta*. By comparing the C-to-U editing sites in human *Homo sapiens* [35,36], we found that five of the 24 C-to-U editing sites in *Macaca mulatta* are conserved in *Homo sapiens* (Figure 5a). The editing levels of these 24 C-to-U editing sites were generally very low and most of these C-to-U editing sites were only detected in a few samples (see Figure 5a). The two C-to-U editing sites on mml-mir-125b-1 and mml-mir-125b-2 were detected in many different tissues (Figure 5a). Similarly, the two conserved C-to-U editing sites in hsa-mir-125b-1/-2 were also identified in multiple human brain [36] and colon tissues [35]. Two other conserved C-to-U editing sites on mml-mir-219-1 and mml-mir-219-2 were mainly identified in brain samples, suggesting their potential functions in brains. Similarly, the two C-to-U editing sites in human hsa-mir-219-1/-2 were only detected in brain tissue samples [36].

The C-to-U editing sites prefer a narrow region from the ninth to eleventh nucleotides of mature miRNAs (Figure 5b). The details of three C-to-U editing sites are shown in Figure 5c–e, respectively, and the reads supporting these sites are shown in Figure 5f,h,i respectively. mml-mir-125b-1_25_C_u is a conserved C-to-U editing sites. The same position of hsa-mir-125b-1 has a significant C-to-U editing event (see Figure 5g) in some human brain samples [36]. The other four conserved C-to-U editing sites are shown in Figure S4.

A previous study reported that a C-to-U editing site on hsa-miR-100-5p is functional because the edited hsa-miR-100-5p represses another target gene SMAD2 that is not a target of the original hsa-miR-100-5p [32]. We found that the same position of mml-miR-100 has a C-to-U editing event in some of our selected samples, but on insignificant editing levels.

### 2.8. Non-Canonical Editing Sites in miRNAs of Macaca mulatta

In addition to the canonical A-to-I and C-to-U editing types, we also identified some non-canonical editing types in miRNAs of *Macaca mulatta* (see Figure 3b). We carefully examined two non-canonical editing types with the largest numbers of editing events, i.e., the 42 G-to-U sites (in Table S12) and the 27 A-to-C sites (in Table S13), in Figures S5 and S6, respectively. As shown in Figure S5a, the editing levels of G-to-U editing sites were generally low. There is no clear positional preference of G-to-U editing sites on mature miRNAs, although position 18 has a slightly more number of G-to-U sites (Figure S5b). Three examples of G-to-U editing sites are shown in Figure S5c–h. One of the G-to-U editing sites, mml-let-7e_18_G_u, was conserved in human *Homo sapiens* [50].

Most (21/27) of A-to-C sites were identified in blood samples (Figure S6a). The editing levels of most A-to-C editing sites (26/27) were lower than 15%, except mml-mir-345_28_A_c (Figure S6a). This site is detected in three samples all with approximately 100% editing levels, suggesting it might be an SNP (Figure S6a). Positions 5 and 18 of mature miRNAs have more numbers of A-to-C editing sites (Figure S6b). Four A-to-C editing sites are shown in Figure S6c–j. As shown in Figure S6e,i, mml-mir-345_28_A_c has an editing level of 99.4% in one of the brain samples selected (SRR1270157).

Except A-to-I and C-to-U editing, the mechanisms of other editing types are largely unknown till now. However, some of these non-canonical types had been reported previously. For examples, we previously noticed that two human miRNAs, hsa-mir-378i and hsa-mir-1260a, carry an A-to-C and a U-to-G editing site, respectively, by integrating the analysis of the genome sequencing profiles from the same cell lines [36]. Several A-to-C and G-to-U editing sites were also reported in another study of human *Homo sapiens* [50]. At least one of the significant G-to-U editing sites identified in *Macaca mulatta* (mml-let-7e_18_G_u) were conserved in *Homo sapiens* [50]. In some plants, U-to-C editing may happen to some mRNAs [33]. All of these results suggest that some non-canonical editing sites may be biologically relevant. However, more work is still needed to further clarify the mechanisms of these non-canonical editing types.

### 2.9. Identified SNPs in miRNAs of Macaca mulatta

**Figure 6:**
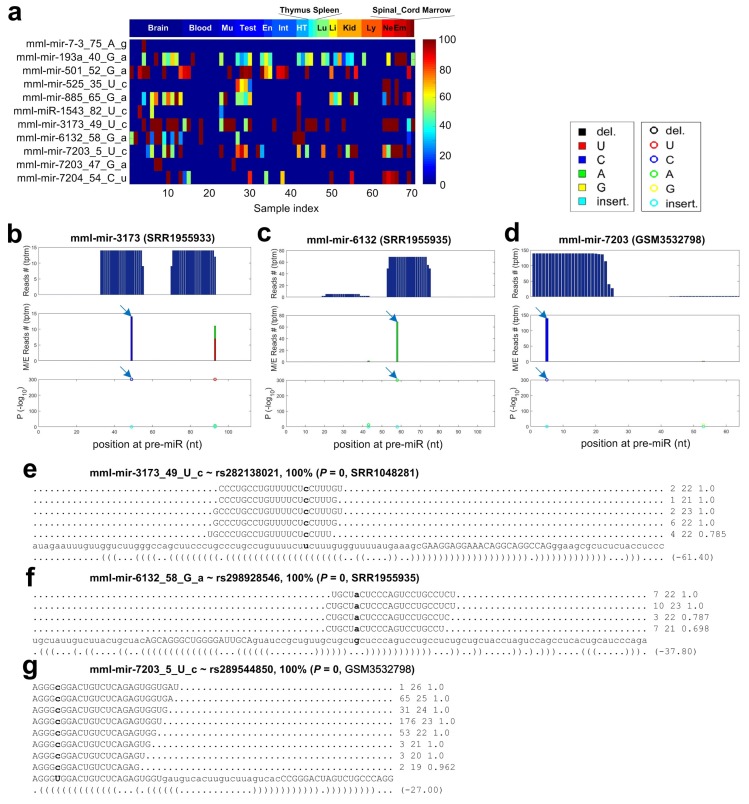
The details of 11 SNPs identified in miRNAs of *Macaca mulatta*. (**a**) The editing/mutation levels of the 11 SNP sites in the 70 selected sRNA-seq profiles. (**b**) The MiRME map of mml-mir-3173 in the bone marrow sample selected (SRR1955933). (**c**) The MiRME map of mml-mir-6132 in the one of the brain samples selected (SRR1955935). (**d**) The MiRME map of mml-mir-7203 in the spinal cord sample selected (GSM3532798). (**e**) The details of mml-mir-3173_49_U_c, i.e., rs282138021, in SRR1955933. (**f**) The details of mml-mir-6132_58_G_a, i.e., rs298928546, in SRR1955935. (**g**) The details of mml-mir-7203_5_U_c, i.e., rs289544850, in GSM3532798.

The 3386 identified M/E sites were compared to SNPs of *Macaca mulatta* reported in dbSNP (v145). The M/E sites that satisfied the following criteria: (i) had the same genomic positions as the SNPs; (ii) had the same nucleotides as the alleles of the SNPs for both the original and changed nucleotides; and (iii) had editing levels of 100% in at least one of the 70 samples selected, were regarded as SNPs. By following these criteria, 11 of the 3386 M/E sites were identified as SNPs (as shown in Figure 6 and Table S14). We also identified two SNP sites in species specific miRNAs using the same criteria (as shown in Figure S7).

Three examples of SNPs in miRNAs are shown in Figure 6b–g. The three SNP sites have editing levels of 100% in the three selected samples (Figure 6e–g). These three SNPs all locate in the mature miRNAs, and two of them (mml-mir-6132_58_G_a and mml-mir-7203_5_U_c) locate in the seed regions (Figure 6b–g). We analyze the potential changes of targets of these two miRNAs due to these two SNPs in the following.

### 2.10. The Potential Functions of M/E Sites Identified in miRNAs of Macaca mulatta

To evaluate the potential functions of the identified M/E sites, we selected 69 M/E sites in the seed regions of mature miRNAs (in Table S15), i.e., 18 A-to-I, 1 C-to-U, 48 Other and 2 SNP sites, and predicted the targets for the original miRNAs and changed miRNAs. We did not find putative targets for the species specific miRNA with an SNP in its seed region. Then, we evaluated the enriched the Gene Ontology (GO) terms and KEGG pathways of the targets of the original and edited miRNAs. The potential targets, and their enriched GO terms in the three major categories, biological process, cellular component, and molecular function, and KEGG pathways of the original and edited miRNAs were then compared to examine the consequences of the M/E events.

**Figure 7:**
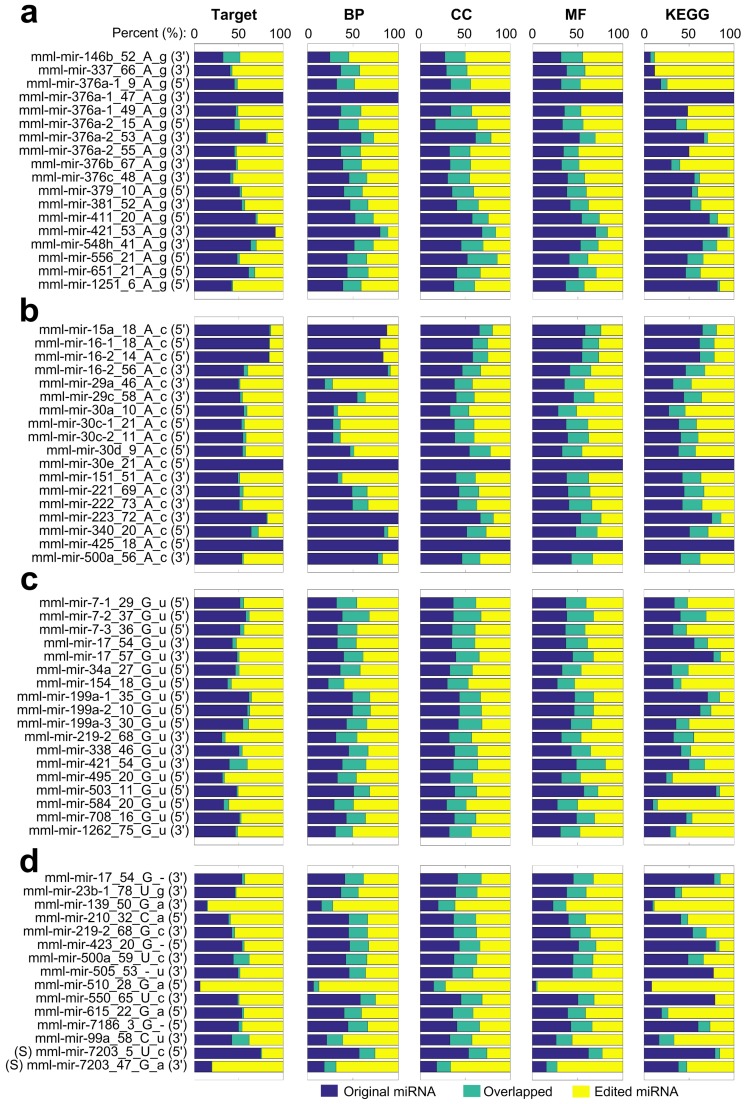
The comparisons of targets, enriched GO terms and KEGG pathways for 69 original and edited/mutated miRNAs whose M/E sites locate in seed regions. Each line started with the MiRME ID of an editing site and the percentage values of predicted targets (in the Target column), enriched GO terms in the categories of biological process (in the BP column), cellular component (in the CC column) and molecular function (in the MF column), and enriched KEGG pathways (in the KEGG column). “(3’)” and “(5’)” after the MiRME ID mean the editing sites locate in the seed regions of mature miRNAs on the 3’ and 5’ arms of pre-miRNAs, respectively. (**a**) The targets, enriched GO terms and KEGG pathways of 18 miRNAs with A-to-I editing sites in their seed regions. (**b**) The targets, enriched GO terms and KEGG pathways of 18 miRNAs with A-to-C editing sites in their seed regions. (**c**) The targets, enriched GO terms and KEGG pathways of 18 miRNAs with G-to-U editing sites in their seed regions. (**d**) The targets, enriched GO terms and KEGG pathways of 13 miRNAs with other non-canonical editing sites and 2 miRNAs with SNPs (marked with “(S)” before names) in their seed regions.

As shown in Figure 7a, the original and 18 A-to-I edited miRNAs only share a small portion of their targets, indicating that these A-to-I editing events severely changed the potential functions of these miRNAs. Consequently, the enriched GO terms and KEGG pathways of the targets of the 18 miRNAs also severely changed due to the A-to-I editing events (Figure 7a).

Similarly, other editing sites in the seed regions and two SNPs in mml-mir-6132 and mml-mir-7203 also lead to severe changes of the targets of the miRNAs, as well as the enriched GO terms of KEGG pathways of their targets (see Figure 7b–d).

## 3. Materials and Methods

### 3.1. Materials and Small RNA Sequencing Profiles

The *Macaca mulatta* animals were raised in Yunnan Key Laboratory of Primate Biomedical Research (LPBR), Kunming, Yunnan, China. The project (No. LPBR 201501003) was approved by the Institutional Animal Care and Use Committee of LPBR on 28 June 2015. We collected twelve samples from the 8 different tissues of two animals, namely muscle (2), liver (1), kidney (2), small intestine (2), large intestine (2), lung (1), spinal cord (1), and occipital lobe (1) without treatments and frozen with liquid nitrogen immediately (Supplementary Table S1). Before RNAs were extracted, these 12 samples were stored at −80 ${}^{\circ }$C. According to the manufacturer’s protocol, we used the Trizol reagent to extract total RNAs from samples. Based on the ratio of the optical density at 260 nm to that at 280 nm (OD260/280), the integrities of RNAs were checked by using an ultraviolet spectrophotometer (Hoefer, MA, USA). The integrities of RNAs were also assessed by electrophoresis in a denaturing formaldehyde agarose gel. The total quantities of RNA samples with OD260/280 between 1.8 and 2.0 were examined. Samples with at least 20 μg were selected for preparation of sRNA sequencing libraries. Then, 20 μg total RNAs dissolved in 35 μL were delivered to the sequencing facility. For small RNA-seq library generation, 1 μg of total RNA was used to prepare a small RNA library according to the TruSeq Small RNA Sample Prep Kit protocol (Illumina, San Diego, CA, USA). Briefly, specific 5’ and 3’ Illumina RNA adapters were sequentially added to small RNA molecules. The 3’ adapter sequence used was TGGAATTCTCGAGTGCCAAGGAACTCC. After reverse transcribed and PCR amplified, adapter-ligated molecules with 15–30 nucleotides long were purified by using gel electrophoresis. The small libraries were then multiplexed and sequenced by using the Illumina HiSeq 2000 sequencer. The qualities of the obtained sRNA profiles were evaluated with the FASTQC program (https://www.bioinformatics.babraham.ac.uk/projects/fastqc/). The obtained small RNA profiles were deposited to NCBI GEO database under the accession number GSE124417.

### 3.2. Identification of Conserved miRNAs

We used a pipeline that was reported previously to identify conserved miRNAs [46,51,52]. Briefly, as shown in Figure S1a, we downloaded all mature miRNA sequences of animal species from the miRBase (v22) [45], and kept unique sequences. Then, these unique miRNA sequences were mapped to the genome of *Macaca mulatta* (Mmul_8.0.1, i.e., GCA_000772875.3, downloaded from the Ensembl Database, https://asia.ensembl.org/Macaca_mulatta/Info/Index) using BLASTN with the parameters of “-m 8 -e 0.1”. Hits with no more than two mismatches were identified. The flanking regions (80 nucleotides downstream and upstream, respectively) of the mapped mature miRNAs were isolated and used to predict secondary structures using the RNAfold with its default parameters. The predicted fold-back structures were examined for the presence of miRNA on the same arm of the hairpin as the known family members from other animals. MIRcheck [53] with its default parameters was used to further evaluate these precursor sequences. The precursors were selected which have ≤2 bulged, ≤5 mismatches, or asymmetrically unpaired nucleotides, and ≤3 continuous mismatches within the mature miRNA. Then, we aligned the obtained 12 sRNA-seq profiles to these pre-miRNA candidates to check the distributions of reads on the pre-miRNAs using the MMFinder pipeline with its default parameters [46]. In total, 992 pre-miRNAs with a clear accumulation of sequencing reads in the mature miRNA regions were called as conserved pre-miRNAs.

The genomic loci of 617 *Macaca mulatta* miRNAs in the miRBase (v22) were downloaded in the GFF3 file format. Because the chromosome names used in the GFF3 file of miRBase were converted to GenBank sequence IDs as defined on https://www.ncbi.nlm.nih.gov/assembly/GCF_000772875.2/#/def. The gene annotation of *Macaca mulatta* (Mmul_8.0.1, i.e., GCA_000772875.3) in the GFF3 file format was downloaded from the Ensembl Database. 1951 loci whose types were annotated as “miRNA” were retrieved from the Ensembl gene annotation file. The 992 pre-miRNAs identified in this study were compared to the reported ones of *Macaca mulatta* in the miRBase (v22) and the Ensembl Database (Mmul_8.0.1) by using BEDTools [54] with the options of “bedtools intersect -wo -s”. There are 1331 pre-miRNAs that are only reported in Ensembl database and do not have mature miRNA annotation. Therefore, we aligned the mature miRNAs in the miRBase (v22) to these 1331 pre-miRNAs and found mature miRNAs for 293 of these 1331 pre-miRNAs. The 1038 remaining pre-miRNAs were not used for identifying mutation and editing sites.

### 3.3. Analysis of the Expression Patterns of Conserved miRNAs

The RPTM values of 2756 mature miRNAs in the 1525 pre-miRNAs were calculated by mapping the sequencing reads to the identified mature miRNAs using a computational pipeline introduced previously [46]. miRNA with average abundances of at least 5 RPTM in the 12 self-generated samples and standard deviation of at least 1 were selected to perform further analysis. The RPTM values of selected miRNAs were applied to the clustergram function in MatLab (Mathworks, Natick, MA, USA) to perform bi-clustering analysis. The RPTM values plus one of selected miRNAs were log-scaled and used to perform Principle Component Analysis (PCA) with the prcomp function in the psych package of the R platform.

### 3.4. Identification of Species Specific miRNAs

We used a pipeline that was reported previously to identify species specific miRNAs [46,52]. We followed the criteria widely used for annotating miRNAs [46,49,55] when identifying species specific miRNAs. As shown in Figure S1c, firstly, to minimize the possibility of false positive predictions caused by low scored nucleotides, i.e., with sequencing scores smaller than 30, we kept raw reads that had no low scored nucleotides in the first 25 nucleotides. Secondly, after removing 3’ adaptor sequences of the remaining reads and redundant sequences, we obtained unique small RNAs. Thirdly, the unique small RNA sequences were aligned to the genome of *Macaca mulatta* (v5) with SOAP2 [56]. We retrieved small RNAs that could be aligned to both strands of the genome. Fourthly, we aligned unique small RNA sequences to known repeats and reported non-coding RNAs (snRNAs, tRNAs, rRNAs, and snoRNAs) by aligning them to databases, Rfam (r11) [57], NONCODE (v3.0) [58], Silva [59], miRBase (r22) [45], and Repbase (r20) [60] using SOAP2 [56] (as shown in Figure S1b). Fifthly, to obtain the sequences that were aligned to above different types of molecules, we retrieved the small RNAs mapped to these molecules, counted and summarized the numbers of reads that were mapped to different types of molecules (as listed in Table S2). Sixthly, we concatenated the alignment results to mRNAs, pre-miRNAs, ncRNAs and repeats to a whole file, and then obtained all unique sequences that could be mapped these molecules. Seventhly, we obtained the sequences that could be aligned to the genome, but not to mRNAs, pre-miRNAs, ncRNAs and repeats. Eighthly, after filtering sequences with limited abundances, we kept unique sequences with at least a total of 10 reads in the 12 sRNA-seq profiles to make sure that the identified species specific miRNAs had enough expression levels. Ninthly, we aligned the sequences obtained above to the genome using SOAP2 [56] and obtained the flanking sequences of the matched loci. Tenthly, we kept the sequences with hairpin structures using RNAfold with its default parameters. Eleventhly, we kept those whose minimal folding energies were smaller than -40 Kcal/mol and had at least 18 paired nucleotides and only one central loop in the hairpin structure. Finally, the unique small RNA reads obtained in the second step were aligned to the obtained hairpin structured putative pre-miRNAs. The distributions of small RNA reads on these putative pre-miRNAs were then obtained using the MMFinder pipeline with its default parameters [46] and examined manually to make sure that mature miRNAs on the 5’ and 3’ arms of the hairpin structure should have a two nucleotide overhang at the 3’ end. Only those putative pre-miRNAs with a good accumulations of sequencing reads on the mature miRNA regions were predicted as species specific miRNAs. In other words, the reads that were originated from the mature miRNAs regions with two nucleotide shifts should account for at least 65% of reads that could be aligned to the same pre-miRNAs.

### 3.5. Identifying Editing and Mutation Sites in miRNAs

In addition to the 12 self-generated sRNA-seq profiles, we selected 58 published sRNA-seq profiles (Table S7) to comprehensively analyze the editing and mutation sites in pre-miRNAs. The selected sRNA-seq profiles were analyzed using the MiRME pipeline [36] with the default settings (as shown in Figure S1d). Briefly, the raw reads were examined to keep the qualified reads of which the sequencing scores of the 25 nucleotides have sequencing scores of 30 or higher. Then, the 3’-adapters in the raw reads (as listed in Table S7) were removed. Then, the unique sequences of the remaining reads, i.e., unique reads, were obtained and the counts of unique reads with more than 18 nucleotides were calculated. The unique reads were aligned to pre-miRNAs using BLASTN with the options of “-S 1 -m 8 -e 0.01” and the reads mapped to pre-miRNAs were retrieved. Then, the reads mapped to pre-miRNAs were aligned to the genome using Bowtie [61] with the options of “-a –best -S -v 1”. Then, the alignments to genome were corrected using the cross-mapping correction algorithm with the default parameters [19]. In the main step, the MiRME algorithm with the default parameters [36] was used to identify the editing and mutation sites from the sequences and structures of pre-miRNAs, the alignments of reads to pre-miRNAs generated by BLASTN, the reads mapped to pre-miRNAs, the alignments of reads against genome generated by Bowtie, and the results of the cross-mapping correction method.

The criteria used to define significant M/E sites include: (i) the relative level of editing is at least 5%; (ii) at least 10 reads support the editing event; (iii) the score threshold of sequencing reads is 30; and (iv) a multiple-test corrected *P*-value of smaller than 0.05. Then, the obtained results of different samples were combined by a separate program in the MiRME package (see the Supplementary Materials). The identified M/E sites were compared to known editing sites in miRNAs of *Homo sapiens*, including the DARNED database [62], the RADAR database [63] and the literature [18,23,24,36,64,65]. Finally, the predicted M/E sites that belonged to A-to-I, C-to-U and Other were manually examined.

As defined previously [36], the significant M/E sites were classified as 8 different categories, i.e., 3’-A, 3’-U, 3’-Other, 5’ site, A-to-I, C-to-U, Other, and Pseudo sites.

To examine the editing levels of miRNAs in different organs or tissues, we grouped the samples of the same tissues as 17 different types of organs or tissues (see Table S7).

The identified M/E sites were also compared to the SNPs reported in the dbSNP (v145). An M/E site was predicted as putative SNP if it satisfies the following criteria: (i) the genomic position of the M/E site is the same as an annotated SNP; (ii) the original and edited nucleotides are the same as the alleles of the SNP; and (iii) the M/E site has editing level of 100% in at least one of the selected samples.

### 3.6. Predicting Putative Targets for the Original and Changed miRNAs

The 3’ UTR sequences of *Macaca mulatta* were downloaded from the Ensembl database (version Mmul_8.0.1). The putative targets of the original and changed, i.e., edited or mutated, miRNAs were predicted with the HitSensor algorithm [66]. Predicted targets with at least seven continuous Watson–Crick matches in the seed regions were maintained in the analysis.

### 3.7. Go and Pathway Analysis for the Original and Changed miRNAs

The GO term and KEGG pathway enrichment of the targets of the original and changed miRNAs were analyzed with KOBAS (version 3.0) [67]. The GO terms and KEGG pathways with multi-test corrected *p*-values smaller than 0.05 were regarded as significant and used for comparisons. Because the GO terms were divided into Biological Process, Cellular Component and Molecular Function, we organized the enriched GO terms into these three categories, and compared the enriched GO terms in these three categories for the original and edited miRNAs, respectively.

## 4. Conclusions

After systematically identifying conserved and species specific miRNAs in *Macaca mulatta* in this study, we identified 479 conserved and 17 species specific pre-miRNAs in this species. Some of the conserved miRNAs show tissue specific expression patterns. We identified 3386 significant M/E sites in miRNAs of *Macaca mulatta* after analyzing 70 sRNA-seq profiles from 17 different types of organs or tissues. These M/E sites include 32 canonical A-to-I and 24 C-to-U editing sites, as well as some non-canonical editing sites. By comparing the identified A-to-I and C-to-U editing sites to results of *Homo sapiens*, we identified 16 conserved A-to-I and five conserved C-to-U editing sites in miRNAs of *Macaca mulatta*. Sixty-seven editing sites and 2 SNPs in the seed regions of miRNAs could severely change the target sets of these miRNAs, which consequently change their potential functions. These results significantly increase our knowledge of miRNAs and their mutation and editing events in *Macaca mulatta*.

## 5. Declarations

### 5.1. Ethics Approval

The *Macaca mulatta* animals were raised in Yunnan Key Laboratory of Primate Biomedical Research (LPBR), Kunming, Yunnan, China. All animal protocols were approved in advance by the Institutional Animal Care and Use Committee of LPBR.

### 5.2. Availability of Data and Materials

The 12 self-generated sRNA-seq profiles of *Macaca mulatta* available at NCBI GEO database with the series accession number GSE124417. The 58 published sRNA-seq profiles were retrieved from the NCBI SRA database under the accession numbers listed in Table S7.

## Supplementary Materials

The following are available online at https://www.mdpi.com/2073-4409/8/7/682/s1, Figure S1: A schematic view of the analysis steps of the sRNA-seq profiles to identify miRNAs and their editing sites in *Macaca mulatta*, Figure S2: The details of 3 of the 16 conserved A-to-I sites in miRNAs of *Macaca mulatta* and *Homo sapiens*, Figure S3: The percentages of nucleotides around the 32 A-to-I editing sites in miRNAs of *Macaca mulatta*, Figure S4: The details of 4 conserved C-to-U editing sites in miRNAs of *Macaca mulatta* and *Homo sapiens*, Figure S5: The details of the 42 G-to-U editing sites identified in miRNAs of *Macaca mulatta*, Figure S6: The details of the 27 A-to-C editing sites identified in miRNAs of *Macaca mulatta*, Figure S7: The details of 2 putative SNP sites identified in novel miRNAs of *Macaca mulatta*, Table S1: The information of 12 sRNA-seq profiles from different tissues of *Macaca mulatta* generated in this study to identify conserved and novel miRNAs, and their editing sites, Table S2: The distributions of small RNAs reads in the 12 sRNA-seq profiles generated in this study in different types of molecules, Table S3: The conserved pre-miRNAs of *Macaca mulatta* identified in our study and reported in the miRBase (v22) and the Ensembl database (version Mmul_8.0.1), Table S4: The normalized abundances of selected conserved miRNAs with enough abundances, Table S5: The miRNAs that are highly expressed in different tissues. Set1 to Set10 correspond to the miRNAs in the Rectangle 1 to 10 in Figure 1b, Table S6: The 17 species specific pre-miRNAs identified in *Macaca mulatta*, Table S7: The information of 70 sRNA-seq profiles from different tissues of *Macaca mulatta* used in this study to identify the miRNA editing sites, Table S8: The 3386 mutation/editing sites with significant editing levels identified in conserved miRNAs of *Macaca mulatta*, Table S9: The 19 mutation/editing sites with significant editing levels identified in species specific miRNAs of *Macaca mulatta*, Table S10: The 32 A-to-I editing sites identified in miRNAs of *Macaca mulatta*, Table S11: The 24 C-to-U editing sites identified in miRNAs of *Macaca mulatta*, Table S12: The 42 G-to-U editing sites identified in miRNAs of *Macaca mulatta*, Table S13: The 27 A-to-C editing sites identified in miRNAs of *Macaca mulatta*, Table S14: The 11 SNP sites identified in miRNAs of *Macaca mulatta*, and Table S15: The 69 M/E sites in the seed regions of mature miRNAs of *Macaca mulatta*.
