# Supplementary material for: Identifying microRNAs and Their Editing Sites in Macaca mulatta
Source: Cells. 2019 Jul 5;8(7):682. doi: 10.3390/cells8070682 (PMC6678584; doi:10.3390/cells8070682)
Supplement: Supplementary file 1 [file cells-08-00682-s001.zip › SupplementaryFigures-v3.pdf]

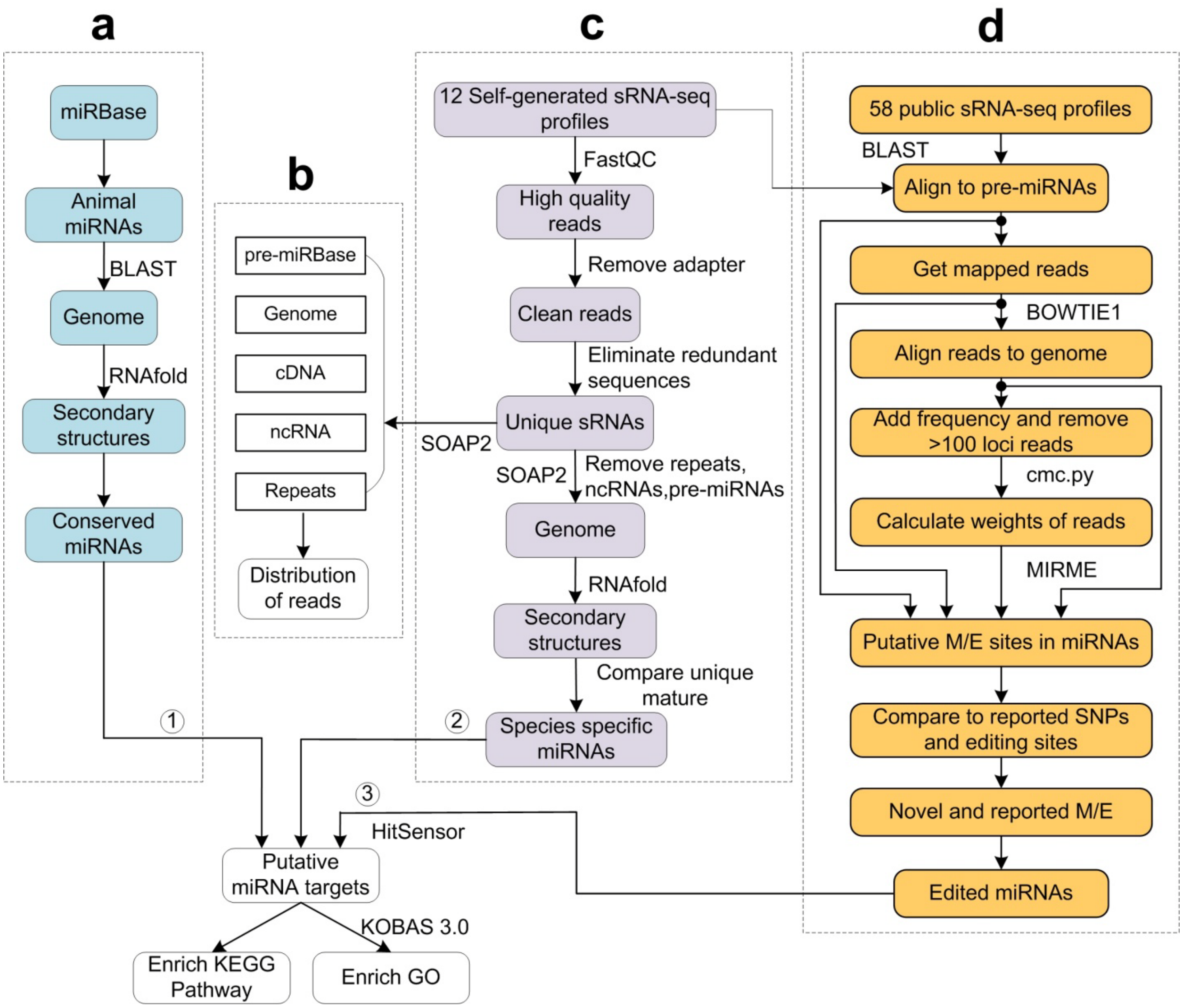

Figure S1. A schematic view of the analysis steps of the sRNA-seq profiles to identify miRNAs and their editing sites in *Macaca mulatta*. The arrows indicate the steps and the related algorithms of the corresponding steps are provided beside the arrows. (a) The computational steps for identifying conserved miRNAs. (b) The computational steps for analyzing the distributions of sRNA sequencing reads that are aligned to different categories of molecules. (c) The computational steps for identifying species specific miRNAs from the 12 self-generated sRNA-seq profiles. (d) The computational steps for identifying mutation and/or editing sites in miRNAs of *Macaca mulatta* from the 12 self-generated and 58 public sRNA-seq profiles. ①, ②,and ③ represent the prediction of putative targets for conserved, species specific and edited miRNAs, respectively, with the HitSensor algorithm. The enriched KEGG pathways and enriched GO terms of the predicted miRNA targets were calculated with KOBAS (v3.0).

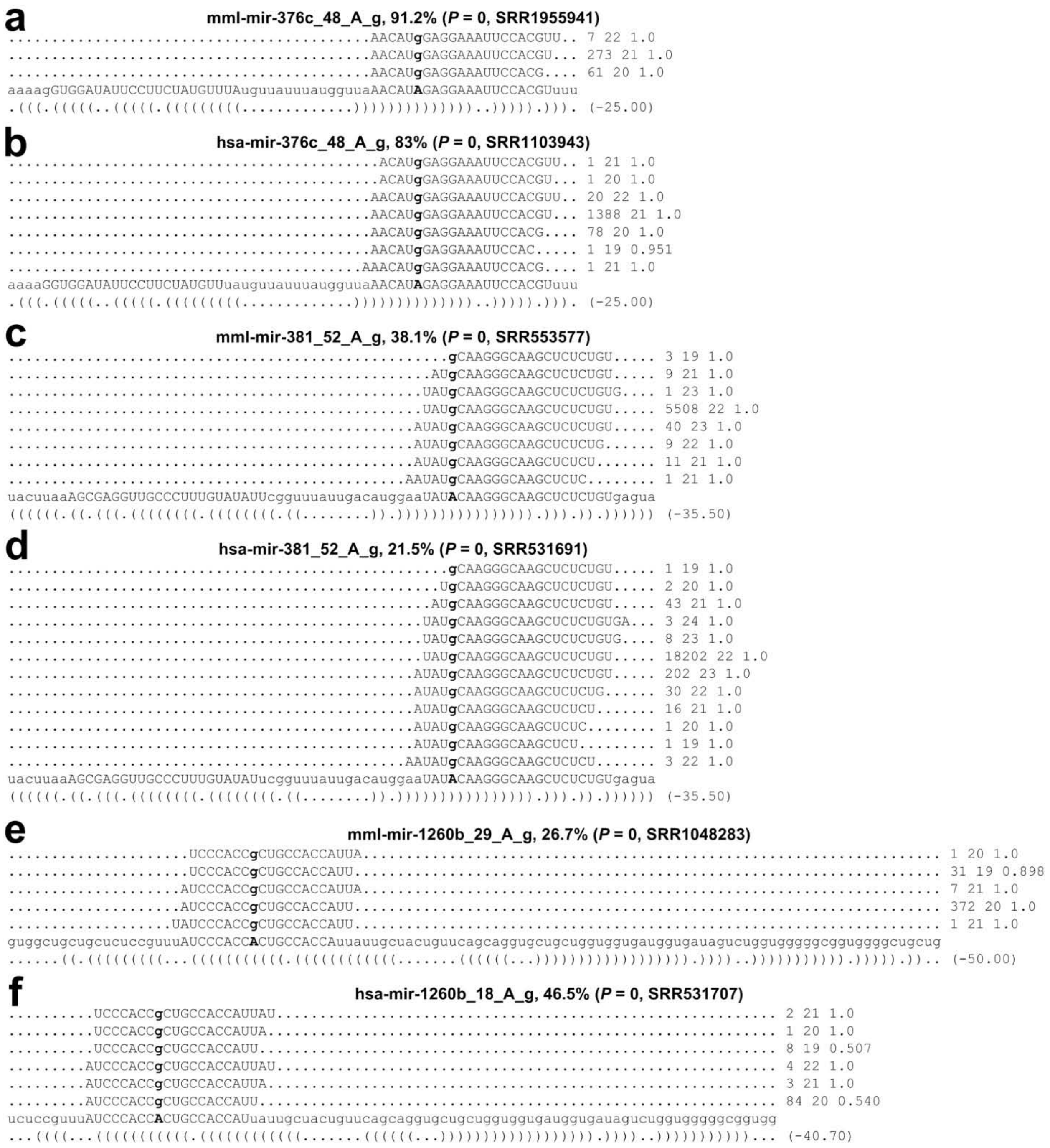

Figure S2. The details of 3 of the 16 conserved A-to-I sites in miRNAs of *Macaca mulatta* and *Homo sapiens*. The results of miRNA editing sites in human *Homo sapiens* were reported previously [1]. (a) The details of mml-mir-376c_48_A_g in the sample of brain pituitary gland selected (SRR1955941). (b) The details of hsa-mir-381_52_A_g in a sample of brain germ cell tumor (SRR531691). (c) The details of mml-mir-381_52_A_g in one of the frontal cortex samples selected (SRR553577). (d) The details of hsa-mir-381_52_A_g in a brain germ cell tumor (SRR531691). (e) The details of mml-mir-1260b_29_A_g in one of the neural cell

samples selected (SRR1048283). (f) The details of hsa-mir-1260b_18_A_g in a brain embryonal tumor sample (SRR531707).

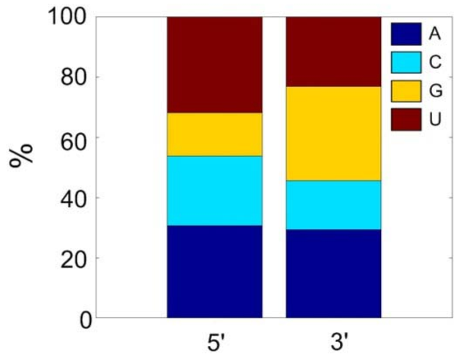

Figure S3. The percentages of nucleotides around the 32 A-to-I editing sites in miRNAs of *Macaca mulatta*.

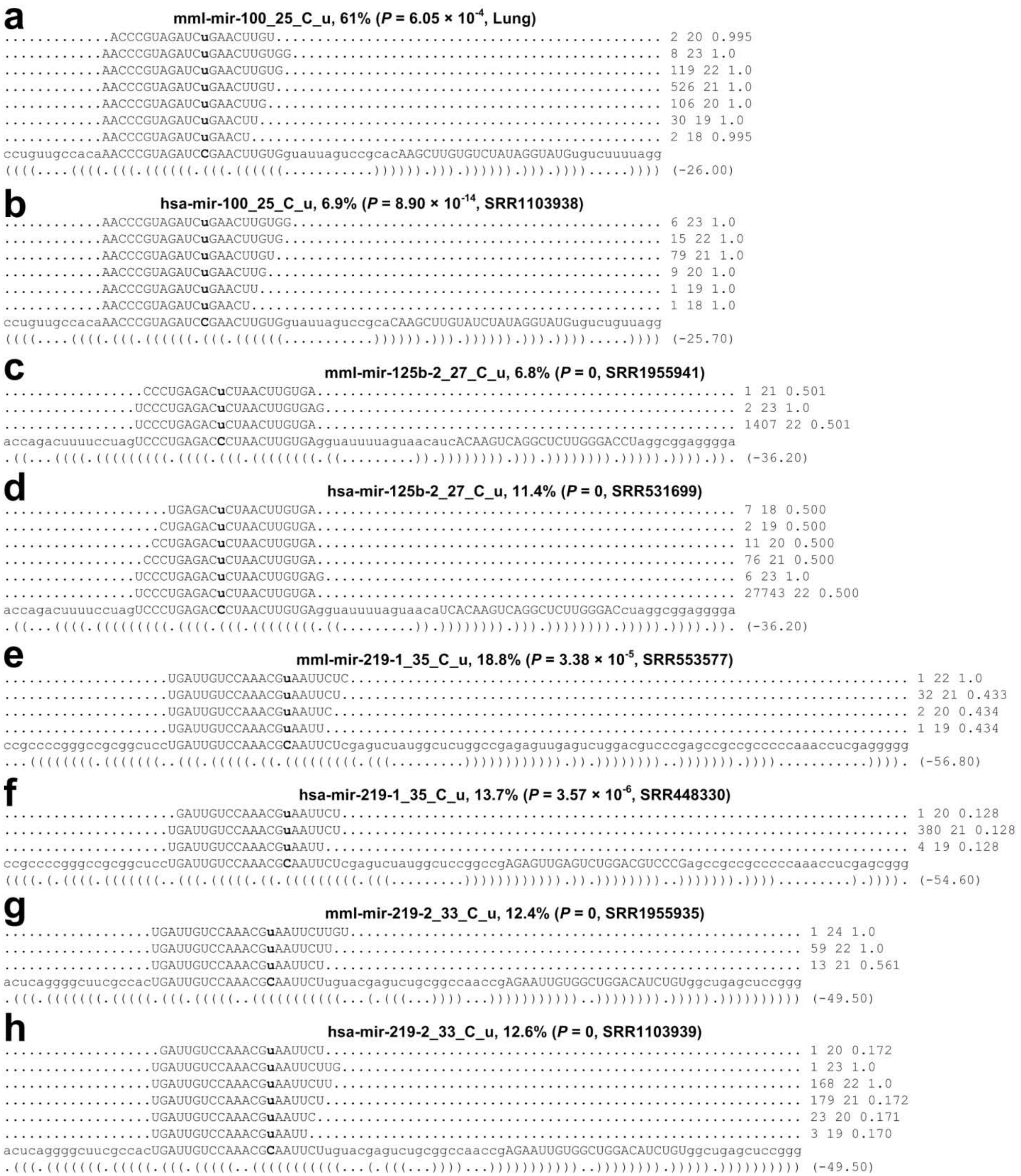

Figure S4. The details of 4 conserved C-to-U editing sites in miRNAs of *Macaca mulatta* and *Homo sapiens*. The results of miRNA editing sites in human Homo sapiens were reported previously [1]. (a) The details of mml-mir-100_25_C_u in one of the lung samples selected (GSM3532792). (b) The details of hsa-mir-100_25_C_u in a prefrontal cortex sample (SRR1103938). (c) The details of mml-mir-125b-2_27_C_u in one of the brain samples selected (SRR1955941). (d) The details of hsa-mir-125b-2_27_C_u in a glioma sample (SRR531699). (e)

The details of mml-mir-219-1_35_C_u in one of the brain samples selected (SRR553577). (f) The details of hsa-mir-219-1_35_C_u in a frontal lobe sample (SRR448330). (g) The details of mml-mir-219-2_33_C_u in one of the brain samples selected (SRR1955935). (h) The details of hsa-mir-219-2_33_C_u in a prefrontal cortex sample (SRR1103939).

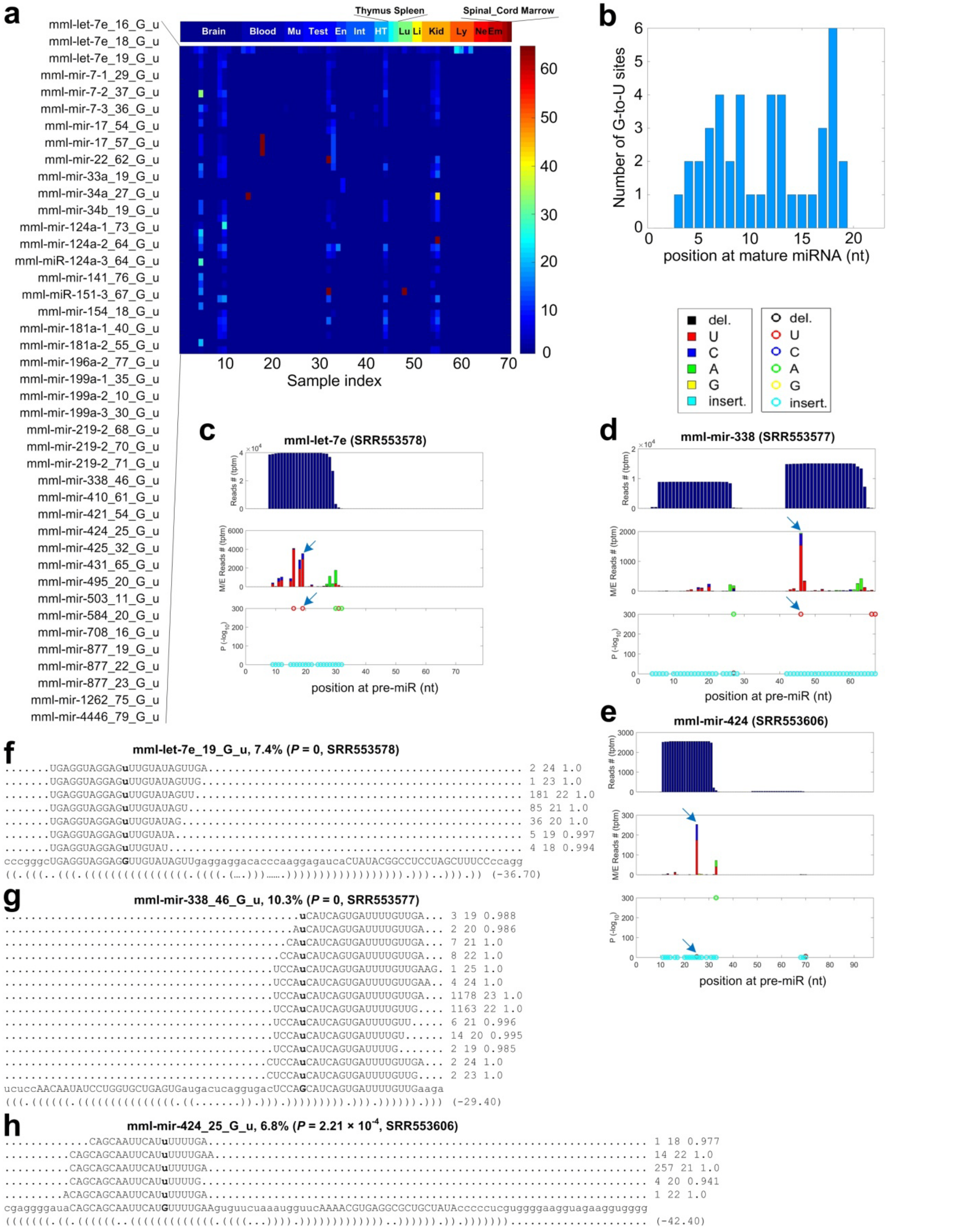

Figure S5. The details of the 42 G-to-U editing sites identified in miRNAs of *Macaca mulatta*. (a) The editing levels of the 42 G-to-U editing sites in the 70 sRNA-seq profiles selected. (b) The

numbers of G-to-U sites at different positions of mature miRNAs. (c) The MiRME map of mml-let-7e in one of the brain samples selected (SRR553578). (d) The MiRME map of mml-mir-388 in one of the brain samples selected (SRR553577). (e) The MiRME map of mml-mir-424 in one of the kidney samples selected (SRR553606). (f) The details of mml-let-7e_19_G_u in SRR553578. (g) The details of mml-mir-338_46_G_u in SRR553577. (h) The details of mml-mir-424_25_G_u in SRR553606.

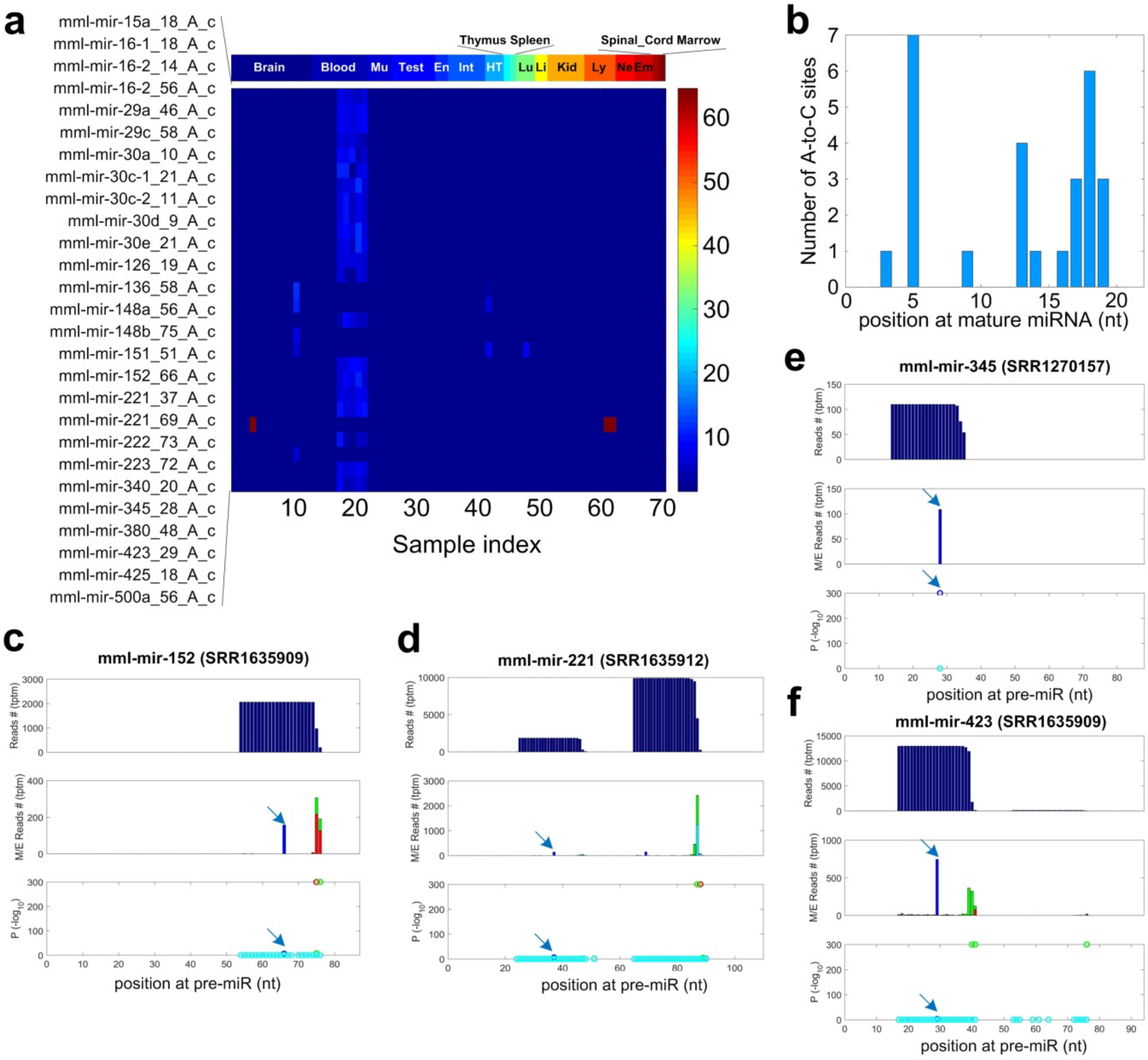

g **mml-mir-152_66_A_c, 7.6% ($P = 6.99 \times 10^{-7}$, SRR1635909)**

```
.....................................................UCAGUGCAUGACcGAACUUGGG............ 74 22 1.0
.....................................................UCAGUGCAUGACcGAACUUGG............. 302 21 1.0
.....................................................UCAGUGCAUGACcGAACUUG.............. 1 20 1.0
ugucccccccggcccAGGUUCUGUGAUACACUCCGACUcgggcucuggagcagUCAGUGCAUGACAGAACUUGGgcccggaaggacc
.((((..((.(((((((((((((.((.((((..((((...(((....)))))))))))).)).))))))))))))).))..)))). (-47.40)
```

■ del.	○ del.
■ U	○ U
■ C	○ C
■ A	○ A
■ G	○ G
■ insert.	○ insert.

h **mml-mir-221_37_A_c, 7.7% ($P = 1.12 \times 10^{-6}$, SRR1635912)**

```
........................ACCUGGCAUACAcUGUAGAUUUCUGU............................................................ 1 26 1.0
........................ACCUGGCAUACAcUGUAGAUUUCU.............................................................. 15 24 1.0
........................ACCUGGCAUACAcUGUAGAUUUC............................................................... 50 23 1.0
........................ACCUGGCAUACAcUGUAGAUUU................................................................ 340 22 1.0
........................ACCUGGCAUACAcUGUAGAUU................................................................. 30 21 1.0
........................ACCUGGCAUACAcUGUAGAU.................................................................. 3 20 1.0
ugaacauccaggucuggggcaugaACCUGGCAUACAAUGUAGAUUUCUGUguucguuaggcaacAGCUACAUUGUCUGCUGGGUUUCaggcuaccuggaaacauguucuc
.((((((((((....(((.((((((((((.((((((((....((((((.....))).)))))))))))).))))))).)))).))))))))))....))))).. (-46.00)
```

i **mml-mir-345_28_A_c, 99.4% ($P = 0$, SRR1270157)**

```
.............GCUGACUCCUAGUCcAGGGCUC..................................................... 80 22 1.0
.............GCUGACUCCUAGUCcAGGGCU...................................................... 31 21 1.0
.............GCUGACUCCUAGUCcAGGGC....................................................... 57 20 0.797094
.............GCUGACUCCUAGUCcAGGG........................................................ 5 19 0.773679
aaacccuaggucgGCUGACUCCUAGUCAAGGGCUCgugguggcuggugggcccugaacgaggguucuggaggccuggguuugaauauc
(((((.(((((..(.((.((((.((..(((((((((.......)))))))))..)).)))).)).)..)))))))))))....... (-37.00)
```

j **mml-mir-423_29_A_c, 5.7% (P = 1.59×10^{-3}, SRR1635909)**

```
................UGAGGGGCAGAGcGCGAGACUUUU...................................................... 196 24 1.0
................UGAGGGGCAGAGcGCGAGACUUU....................................................... 1425 23 1.0
................UGAGGGGCAGAGcGCGAGACUU........................................................ 125 22 1.0
................UGAGGGGCAGAGcGCGAGACU......................................................... 28 21 1.0
................UGAGGGGCAGAGcGCGAGAC.......................................................... 3 20 1.0
................UGAGGGGCAGAGcGCGAGA........................................................... 3 19 0.99713
auaaaggaaguuaggcUGAGGGGCAGAGAGCGAGACUUUucuauuuuccaaaAGCUCGGUCUGAGGCCCCUCAGUcuugcuuccuaccccgcgc
....(((((((.(((((((((((..(((.((((.(((((........)))))))).)))...))))))))))).))))))))......... (-48.60)
```

Figure S6. The details of the 27 A-to-C editing sites identified in miRNAs of *Macaca mulatta*. (a) editing levels of the 27 A-to-U editing sites in the 70 sRNA-seq profiles selected. (b) The numbers of A-to-C sites at different positions of mature miRNAs. (c) The MiRME map of mml-mir-152 in one of the brain samples selected (SRR1635909). (d) The MiRME map of mml-mir-221 in one of the lung samples selected (SRR1635912). (e) The MiRME map of mml-mir-345 in one of the brain samples selected (SRR1270157). (f) The MiRME map of mml-mir-423 in one of the brain samples selected (SRR1635909). (g) The details of mml-mir-152_66_A_c in SRR1635909. (h) The details of mml-mir-221_37_A_c in SRR1635912. (i) The details of mml-mir-345_28_A_c in SRR1270157. (j) The details of mml-mir-423_29_A_c in SRR1635909.

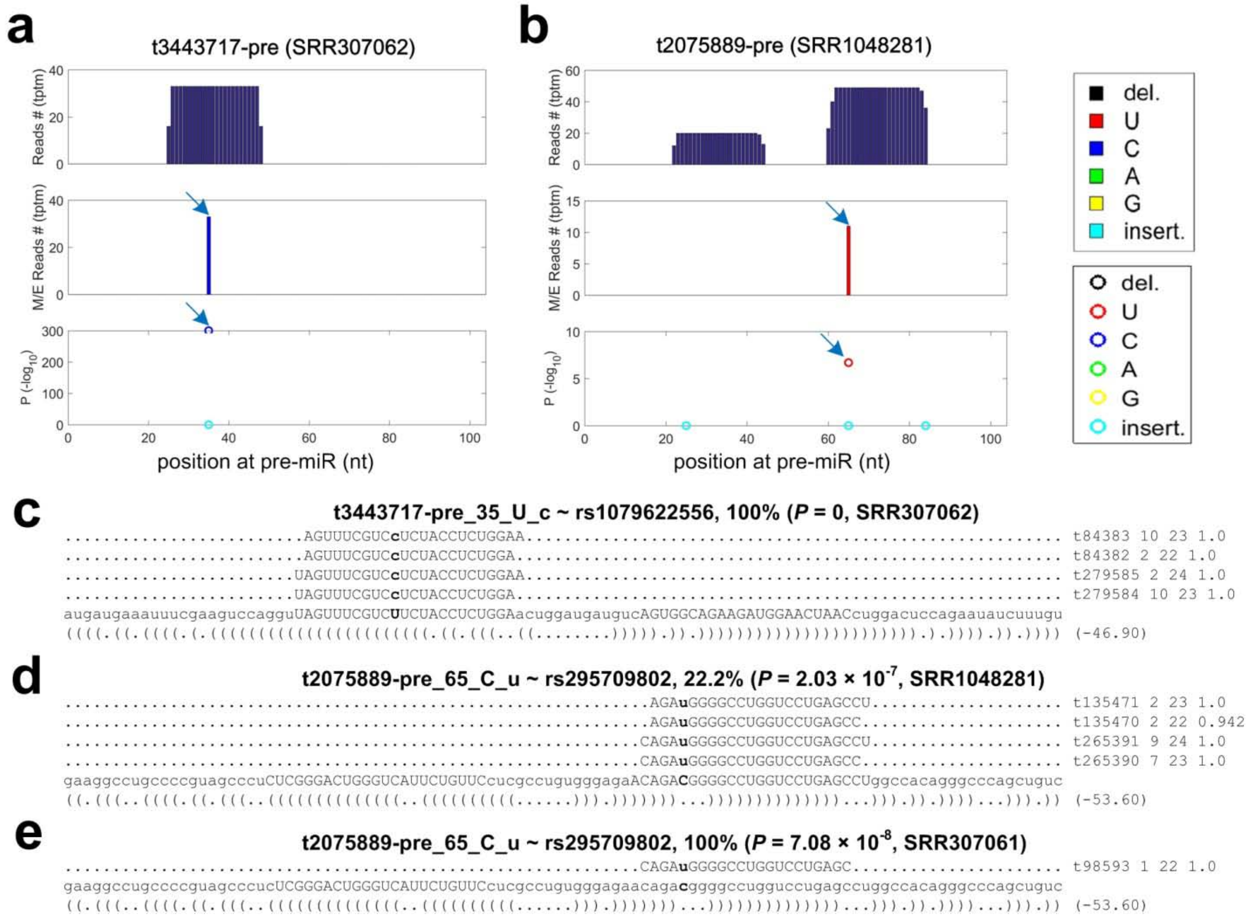

Figure S7. The details of 2 putative SNP sites identified in species specific miRNAs of *Macaca mulatta*. (a) The MiRME map of t3443717-pre in one of the embryonic stem cell samples selected (SRR307062). (b) The MiRME map of t2075889-pre in one of the embryonic stem cell samples selected (SRR1048281). (c) The details t3443717-pre_35_U_c, i.e., rs1079622556 in SRR307062. (d) The details of t2075889-pre_65_C_u, i.e., rs295709802 in SRR1048281. (e) The details of t2075889-pre_65_C_u, i.e., rs295709802 in SRR307061.

Reference

[1] Zheng, Y.; Ji, B.; Song, R.; Wang, S.; Li, T.; Zhang, X.; Chen, K.; Li, T.; Li, J. Accurate detection for a wide range of mutation and editing sites of microRNAs from small RNA high-throughput sequencing profiles. Nucleic Acids Research 2016, 44, e123.
